# A Novel Survey Platform in the Age of COVID-19 to Increase Accuracy and Adoptability While Reducing Selection Bias

**DOI:** 10.1093/geroni/igaa057.3415

**Published:** 2020-12-16

**Authors:** Dinesh Mendhe, Stephanie Bergren, XinQi Dong

**Affiliations:** Rutgers University, New Brunswick, New Jersey, United States

## Abstract

Given the ongoing COVID-19 pandemic, secure and distanced data collection platforms are critical for reaching vulnerable populations. Commonly used electronic data collection systems lack a myriad of critical features, including a modern technology stack, new data encryption and security standards, study workflows, and reporting algorithms. Moreover, these systems do not have multilingual mapping functionalities of survey and consent forms. All of these components ultimately increase selection bias while simultaneously reducing the security and quality of the response data. In order to directly address the aforementioned issues, we have developed a multilingual and highly secure data management platform. Our application is built using stable, tested, and modular programming frameworks and design patterns targeted at accommodating intricately complex structures of polyglot mapping, large volume of data, encryption and granular user authorization. The statistical accuracy along with the multilingual mapping are the core highlights of this system. The multilingual function of this platform has the ability to eliminate selection biases while creating a well-balanced cross-section of society. Modern survey design workflows and validation checks ultimately prevent data loss and help reduce data collection errors. The platform design was initiated in April 1, 2020 and has been pilot tested for use in multilingual populations. The currently active application version of the system is capable of supporting in-person and telephone interviews, emailing survey links to every registered participant, building family tree architecture, and online consent management. This platform also has built-in report functionality. Additional features are being explored to improve study coordination and monitoring.

